# Supplementary material for: Evaluation of antibiotic prescribing for the treatment of male community-acquired urinary tract infections using reimbursement data
Source: PLoS One. 2025 Jul 28;20(7):e0327197. doi: 10.1371/journal.pone.0327197 (PMC12303348; doi:10.1371/journal.pone.0327197)
Supplement: Attachment — Submitted filename: Response_to_Reviewers_auresp_2.docx [file pone.0327197.s004.docx]

PONE-D-24-41708R1

Evaluation of antibiotic prescribing for the treatment of male community-acquired urinary tract infections using reimbursement data

PLOS ONE

**Reviewer #1:** I sincerely thank the authors for taking into account all the comments made by the various reviewers. The manuscript is improved, and the results more robust. I would like to emphasize once again the contribution of these results in relation to male urinary tract infections, which are much less studied than female urinary tract infections.

**Reviewer #3:** The manuscript improved with revisions, however I still find some points questionable.

- At the editor´s discretion, some more changes might be useful. I think that although the French databases allow, in theory, to go back to the prescriber, researchers are usually not allowed to do that. Even if they are (e.g. because who is doing the study is the same group of people that decides on further training of individual physicians), a research study normally does not aim to go back to individual prescribers (or groups of them) for retraining. This aim is more consistent with a clinical audit. Therefore, again, I would suggest you to rephrase referring to an aim that is consistent with the results of your analyses, i.e. I would refer to the identification of areas of improvement or similar, which are, as pointed out in your summary of findings, antibiotic type and duration.

Response: We modified our aim as suggested by Reviewer 3 : “The aim of the study was to assess GPs’ adherence to French recommendations and to identify areas for improvement in antibiotic prescribing practices, particularly regarding antibiotic choice and treatment duration, by characterizing groups of GPs with similar prescribing patterns.”

- The authors pushed back some of my suggestions. This is fine to me, however, after reading the revised manuscript again, I still think that a more international contextualisation of male UTI treatment would be important, given the many observational studies on the topic published internationally. I agree that comparing guidelines was not an objective of the study. However, the discussion of a manuscript normally broadens the perspective comparing what was found to what is already known. As the explicit focus was on guideline adherence, a more international perspective on what in other contexts might be considered appropriate and why in other context GPs adhere or do not adhere to guideline recommendations would be useful and would make the manuscript more interesting for an international readership.

Response: We agree that a broader international perspective would enrich the discussion. However, our study did not investigate the underlying reasons why some GPs adhere to national recommendations while others do not. These reasons are likely multifactorial and context-dependent, and would require qualitative studies or targeted surveys, which were beyond the scope of our work.

We have added a section in the Discussion highlighting the differences in the diagnosis and management of male urinary tract infections across countries. These variations make it difficult to compare guideline adherence between healthcare systems.

- Lastly, I realised that the results of the urine culture were not available. What I understand here is that you were not provided with data on urine culture positivity (as S1 table suggests). What I don´t understand is how the authors assessed if the "documented" antibiotic was appropriate or not. This would need, in my opinion, further explanation in the methods section.

Response: We were not able to assess whether the prescribed treatment matched the antibiotic susceptibility test results, as urine culture data were not available. However, resistance rates in our region are low (with approximately 4% of ESBL-producing strains, which are often resistant to cotrimoxazole, third-generation cephalosporins, and fluoroquinolones). Based on these epidemiological data, we acknowledge that, in a small proportion of cases, physicians may have been unable to prescribe a treatment fully aligned with French recommendations. Nevertheless, this cannot fully explain why only 70% of prescriptions were in accordance with the guidelines. This point is addressed in the Discussion section, lines 304–309.

We explained in the Methods section that antibiotic treatment was evaluated based on the French national recommendations. We have now added that this evaluation was performed as antibiotic susceptibility test results were not available.
